# MRI-based radiomics model for differentiating focal cortical dysplasia from dysembryoplastic neuroepithelial tumor in epileptic children

**DOI:** 10.3389/fneur.2025.1658440

**Published:** 2025-11-13

**Authors:** Xinyi Yang, Shuang Ding, Zhongxin Huang, Xiangmin Zhang, Jinhua Cai

**Affiliations:** Department of Radiology, Children’s Hospital of Chongqing Medical University, Chongqing, China

**Keywords:** focal cortical dysplasia, dysembryoplastic neuroepithelial tumor, epilepsy, radiomics, magnetic resonance imaging

## Abstract

**Objective:**

Focal cortical dysplasia (FCD) and dysembryoplastic neuroepithelial tumor (DNET) are two major causes of intractable epilepsy, often with confusing imaging findings. This study aimed to develop magnetic resonance imaging (MRI)-based radiomics models for preoperative differentiation between FCD and DNET.

**Methods:**

This study included 169 patients who underwent epilepsy surgery and were pathologically diagnosed with FCD (*n* = 96) or DNET (*n* = 73). Conventional brain T1-weighted (T1WI), T2-weighted (T2WI) and T2-fluid-attenuated inversion recovery (T2-FLAIR) images were acquired from all cases. The whole dataset was randomly divided into a training set and a test set at a ratio of 3:1. PyRadiomics software was used for feature extraction and selection. The final features were determined using the least absolute shrinkage and selection operator (LASSO) algorithm. A support vector machine (SVM) was used to establish radiomics models based on individual sequences or fusion of these sequences. The performance of each model was evaluated by the area under the receiver operating characteristic curve (AUC), and the optimal model was also compared with the radiologists’ assessment results.

**Results:**

The fusion radiomics model exhibited the best performance in differentiating between FCD and DNET, with an AUC of 0.894 (95% CI: 0.799–0.968) and an accuracy of 82.0%, which were superior to the individual models based on T1WI, T2WI, or T2-FLAIR images. In addition, the diagnostic performance of the fusion radiomics model was superior to that of the junior radiologist and comparable to that of the senior radiologist.

**Conclusion:**

The fusion radiomics model based on multi-sequence MRI can successfully differentiate FCD from DNET preoperatively, which contributes to appropriate surgical planning and satisfactory treatment outcomes.

## 1 Introduction

Focal cortical dysplasia (FCD) and dysembryoplastic neuroepithelial tumor (DNET) are the most common causes of medically intractable epilepsy in children (1–3). They both require surgical resection, which can help reduce epileptic seizures and improve cognitive outcomes. Nonetheless, previous studies have shown that the postoperative recurrence rate of seizures varies greatly between patients with FCD and DNET. After complete resection, about 80% of patients with DNET will no longer experience seizures, whereas 50% of patients with FCD will still develop seizures of varying degrees (4, 5). Research suggests that blurred histological boundaries of FCD lesions should be taken into account for the surgical resection. Therefore, electroencephalography is often recommended to determine the extent of cortical excision. In contrast, complete surgical resection of DNET and epileptogenic zone (EZ) is thought to be sufficient and effective for preventing postoperative seizures (6, 7). Given the different surgical strategies and prognosis between FCD and DNET, preoperative differential diagnosis is of great importance.

Previous studies have shown that the clinical manifestations of FCD and DNET are similar except that epilepsy occurs earlier in children with FCD than in children with DNET, which is not sufficient to differentiate between the two diseases (8). Although positron emission tomography (PET) with high sensitivity can help identify FCD and DNET, it tends to lack specificity to metabolic decline emerging in the interictal stage (9). At present, magnetic resonance imaging (MRI) has played an important role in the detection and diagnosis of FCD and DNET. Despite many MR scanning techniques and post-processing methods, the imaging features such as the location, shape and signal changes of the two diseases are similar, which may lead to misdiagnosis. To sum up, it is still difficult to differentiate FCD from DNET by the existing imaging methods. Therefore, it is essential to develop a more precise and impartial diagnostic technique to differentiate between the two diseases.

Radiomics generally aims to extract quantitative and ideally reproducible information from diagnostic images, including complex patterns that are difficult for the human eye to recognize or quantify (10–12). By using the radiomics features, the radiomics models can be established to achieve predictions, judgments, and differential diagnosis of diseases (13). A support vector machine (SVM) is a powerful classification algorithm that can estimate the classification probabilities and control complexity, which has been widely used in the field of neuroimaging (14–16), and also applied in related research on FCD (17). In addition, the least absolute shrinkage and selection operator (LASSO) is a regularization technique for minimizing the number of non-zero elements and producing a unique solution (18), which is often used to solve the problem of large sets of radiomics features derived from a relatively small sample size. Previously, it has been verified that LASSO combined with SVM has the best performance in differentiating diseases (19). Therefore, it is expected that the combination of the two can be used to differentiate FCD from DNET.

Therefore, this study aimed to develop radiomics models based on individual sequences (T1WI, T2WI, and T2-FLAIR) or fusion of these sequences for differentiating FCD from DNET, and compare their diagnostic performance. We hope to provide a new high-performance method for preoperative differentiation of FCD and DNET in children with drug-resistant epilepsy.

## 2 Methods

### 2.1 Participants

**Figure 1:**
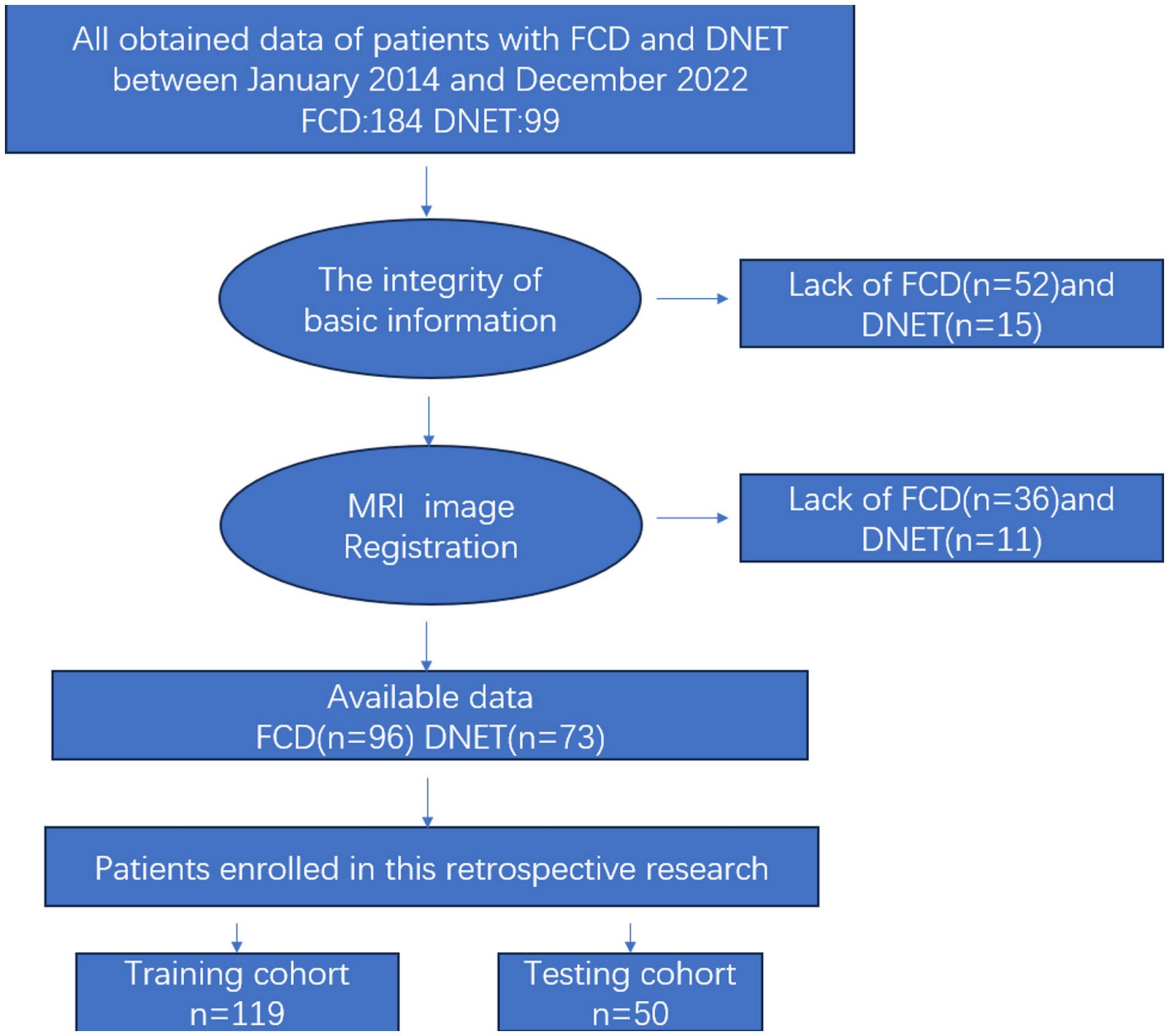
Patient selection flowchart. The subjects were selected according to the inclusion criteria and exclusion criteria. FCD, focal cortical dysplasia; DNET, dysembryoplastic neuroepithelial tumors.

This retrospective study was approved by the Institutional Review Board of our hospital and the requirement to obtain written informed consent was waived. All participants were from the our hospital between January 2014 and December 2022. Inclusion criteria were as follows: (1) Patients diagnosed with epilepsy in accordance with the International League Against Epilepsy (ILAE) Guidelines for Classification and Diagnosis of Epilepsy (20), (2) those undergoing epilepsy surgery and pathologically diagnosed with FCD or DNET, pathological types of FCD include: type I and type II (specifically, subtypes Ia, Ib, Ic, IIa, and IIb according to the ILAE classification), (3) those receiving preoperative non-invasive evaluation, including long-term video-EEG, conventional MRI and PET-CT. Exclusion criteria included: (1) Patients with incomplete clinical data, (2) those with low imaging quality, (3) those with FCD who had any concomitant lesions, or (4) those with recurrent lesions or a history of treatment. The flowchart of patient selection is shown in Figure 1. The clinical data including gender, age, age at seizure onset, type of seizure (based on the new operational classification by the ILAE), past medical history (febrile convulsions, encephalitis, perinatal brain injury, and traumatic brain injury) and kinds of anti-seizure medication administered were recorded.

### 2.2 MRI datasets and preprocessing

All patients were scanned by a 3.0 T scanner (Achieva 3.0 T TX, Philips, Holland) with an 8-channel head coil, with the following scanning parameters: T1WI: repetition time (TR) = 2,000 ms and echo time (TE) = 20 ms; T2WI: TR = 3,500 ms and TE = 80 ms; T2-FLAIR: TR = 8,000 ms and TE = 125 ms. For all images, the field of view (FOV) was 230 × 191 × 143 mm, and slice thickness was 5.0 mm with an inter-slice spacing of 1.0 mm.

For image segmentation, the images of each subject were first aligned to T2-FLAIR using the FSL Version 5.0.9.^1^ Then they were loaded into an open-source image processing software ITK-SNAP (version 4.0.0, http://www.itksnap.org) in DICOM format. The 3D volume of the lesion was manually delineated layer by layer along its contour on T1WI, T2WI, and T2-FLAIR images by two radiologists with 8- and 5-year experience who were independently blinded to the clinical data of patients. During the delineation process, the following principles were strictly followed: (1) Boundary determination: The ROI is strictly delineated along the edge of the lesion, and the adjacent normal brain tissue is excluded as much as possible. (2) Image quality consideration: During the delineation process, actively avoid regions with significant volume effects, motion artifacts, and susceptibility artifacts that may affect the accuracy of the signal. (3) Multi-sequence collaborative judgment: The radiologist refers to the registered images of multiple sequences (such as T1WI, T2WI, and T2-FLAIR) and comprehensively determines the lesion range, ensuring that the ROI is spatially consistent across different sequences. (4) Dispute resolution mechanism: If the two radiologists have a disagreement on the delineation of a certain ROI, an experienced senior radiologist with 15 years of experience in central nervous system imaging diagnosis will arbitrate. This expert will review the registration quality and segmentation consistency of multiple sequence images one by one, and ultimately determine the ROI range to ensure that all subjects’ lesion areas are accurately and consistently covered. The intra- and interobserver agreement was assessed using the intraclass correlation coefficient (ICC), and ICC > 0.75 was considered good agreement.

In terms of image preprocessing, we have indeed carried out several steps to enhance data quality and feature stability, which include: (1) Image resampling (Resampling): All images are uniformly resampled to isotropic resolution (the default value is 1 × 1 × 1 mm^3^) to eliminate the influence of resolution differences among the original images on feature extraction. (2) Grayscale Discretization (Discretization): The grayscale values of the image are discretized using a fixed bin width (the default bin width is 25 HU) to reduce noise and enhance the consistency of features. (3) Image Filtering (Filtering): The default filter set of PyRadiomics was applied, including: Gaussian Laplacian (LoG) filtering and wavelet filtering. Other image enhancement filters (such as gradient, square, etc.). (4) Image mask processing: Before feature extraction, all the regions of interest (ROI) were delineated by experienced radiologists and the masks were ensured to be aligned with the images. All these preprocessing steps were completed using the default parameters of PyRadiomics. The specific settings can be referred to the IBSI guidelines and the official documentation of PyRadiomics. The whole dataset was randomly divided into a training set and a test set at a ratio of 3:1. All the cases in the training set were used to train the diagnostic model, whereas all the cases in the test set were used to independently evaluate the performance of the model.

### 2.3 Feature extraction and feature selection

PyRadiomics software (version 3.0, http://readthedocs.org/projects/PyRadiomics/) was used for feature extraction and feature selection. This tool strictly follows the standards of the Imaging Biomarker Standardization Initiative (IBSI), ensuring the standardization and reproducibility of feature calculations. Finally, a total of 1,130 features were extracted and categorized into seven major types: (1) 18 first-order statistics; (2) 14 shape-based; (3) 24 gray level co-occurrence matrix (GLCM); (4) 16 gray level run length matrix (GLRLM); (5) 16 gray level size zone matrix (GLSZM); (6) 5 neighboring gray tone difference matrix (NGTDM); (7) 14 gray level dependence matrix (GLDM). After normalization, feature screening was further performed using the machine learning algorithm provided by the sklearn package.

### 2.4 Model training and validation

**Figure 2:**
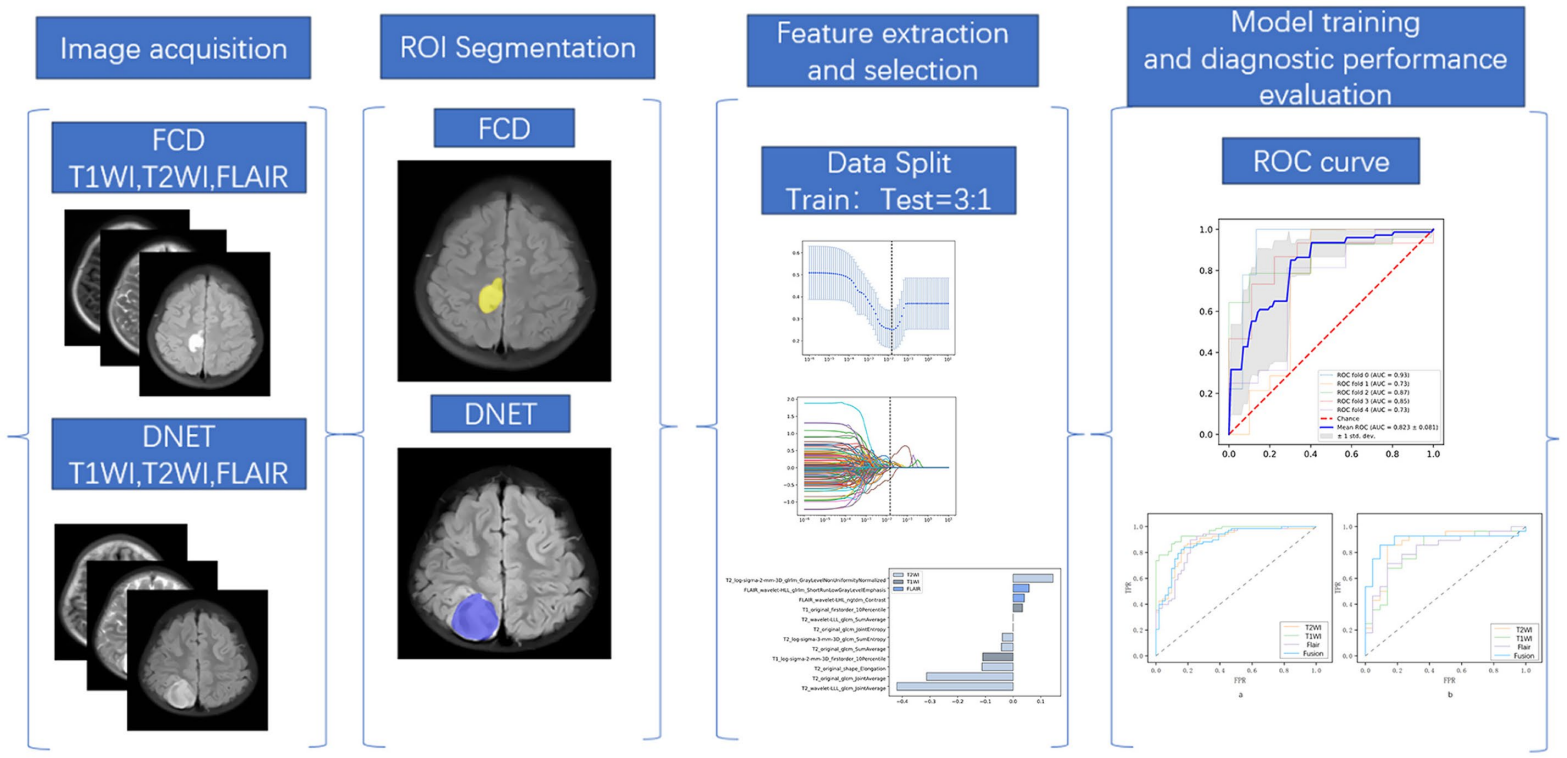
Workflow of the radiomics analysis. The workflow of the radiomics analysis was as follows: Image acquisition, ROI segmentation, feature extraction, feature selection, model training, and diagnostic performance evaluation.

The features with no significant difference between the two groups were filtered by the two-sample *t*-test, and the optimal features were determined using the LASSO algorithm with 5-fold cross-validation. Twenty-five, 8 and 14 feature subsets were extracted from the T1WI-, T2WI- and T2-FLAIR-based models, respectively. Then the above process was repeated to further screen the fusion features of the three, and a total of 12 fusion features were obtained finally. SVM was used for the classification of features. In the training set, GridSearchCV (CV = 5, namely, 5-fold cross-validation) was used to optimize the hyperparameters of the model to reduce its training error and generalization error. The diagnostic efficacy of the model was assessed through the mean area under the receiver operating characteristic (ROC) curve (AUC), sensitivity, specificity, and accuracy. The workflow of data preparation and radiomics analysis is shown in Figure 2.

### 2.5 Radiologists’ assessment

To compare the performance of the radiomics model with the radiologists’ assessment results in differentiating FCD from DNET, two radiologists with 8- and 5-year experience who were blinded to the clinical and pathological data were asked to differentiate FCD from DNET according to the sequences (T1WI, T2WI, and T2-FLAIR). Then the sensitivity and specificity of the radiomics model and the radiologists’ assessment results were compared.

### 2.6 Statistical analysis

Statistical analysis was performed using SPSS26.0. Measurement data (age, age of seizure onset) was expressed as median [interquartile range (IQR)]. Independent sample *t*-test (if homogeneity of variance was met) or Welch’s *t*-test (if heterogeneity of variance was met) was used for comparison between groups. Count data (gender, seizure type, past medical history and Number of anti-seizure medication.) were expressed as the number of cases (percentage) [*n* (%)], and comparison between groups was performed using the Chi-square test or Fisher’s exact test (when the expected frequency was <5). *p* < 0.05 was considered statistically significant.

## 3 Results

### 3.1 Patient characteristics

A total of 169 patients with FCD (*n* = 96) or DNET (*n* = 73) were included, with more males than females in both FCD and DNET groups. Among the 96 FCD patients, the histological subtypes were distributed as follows: type Ia (*n* = 8), type Ib (*n* = 11), type Ic (*n* = 9), type IIa (*n* = 32), and type IIb (*n* = 36). The median age at seizure onset in the FCD group was higher than that in the DNET group. 59 (61.5%) patients in the FCD group and 37 (50.7%) patients in the DNET group administer more than three kinds of anti-seizure medication. The gender, age at seizure onset, type of seizure, past medical history, and kinds of anti-seizure medication administered had no significant differences between the FCD and DNET groups (Table 1).

**Table 1:** Clinical characteristics of patients.

variable	FCD (96)	DNET (73)	stat	*p*
Man/female, *n*	61/35	50/23	0.451	0.502
Age (month), median (IQR)	96(54,120)	72(36,132)	1.024	0.307
Age of seizure onset (month), median (IQR)	84(48,120)	72(36,120)	0.320	0.749
Seizure type, *n* (%)			0.599	0.741
FAS	22(23.0)	15(20.5)		
FIAS	35(36.4)	24(32.9)		
FBTCS	39(40.6)	34(46.6)		
Past History, *n* (%)				0.999
None	67(69.8)	56(76.7)		
Febrile convulsions	21(21.9)	13(17.8)		
encephalitis	2(2.1)	2(2.7)		
perinatal brain injury	5(5.2)	1(1.4)		
traumatic brain injuries	1(1.0)	1(1.4)		
Number of anti-seizure medication, *n* (%)				0.999
1	5(5.2)	7(9.6)		
2	32(33.3)	29(39.7)		
≥3	59(61.5)	37(50.7)		

Data are represented as median (interquartile range) or *n* (%). FCD, focal cortical dysplasias; DNET, dysembryoplastic neuroepithelial tumor. FAS, focal aware seizures; FIAS, focal impaired awareness; FBTCS, focal to bilateral tonic–clonic seizures.

### 3.2 Diagnostic performance of radiomics models

**Figure 3:**
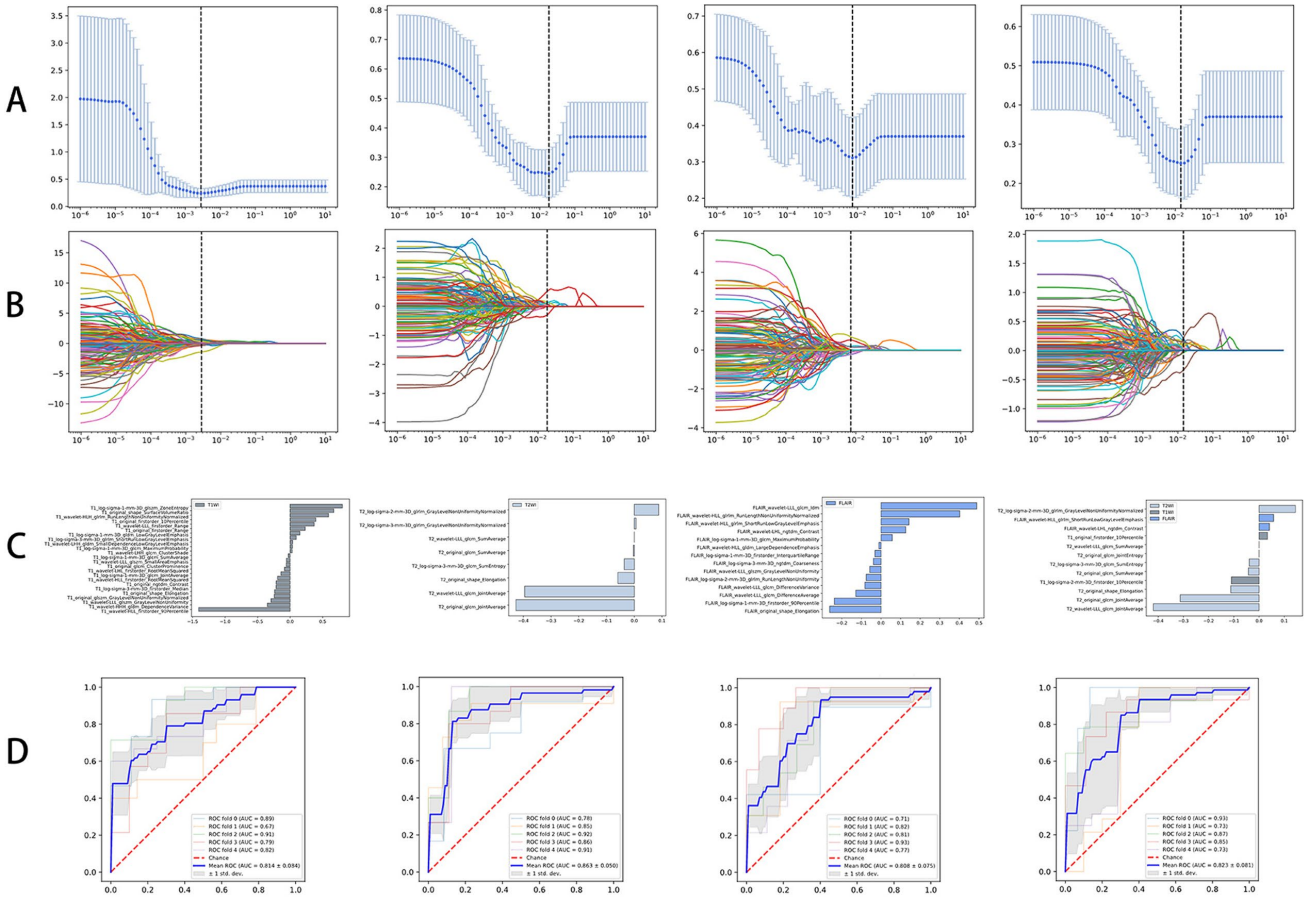
Statistical selection process of radiomic features with LASSO regression. **(A,B)** The decreasing path of mean square deviation and characteristic coefficient with the change of *λ* value. **(C)** Features related to the optimal value were further reserved with respective coefficients to build the radiomics signature model. **(D)** ROC curve were cross-validated with hyperparameters on the training set in individual and fusion model.

**Figure 4:**
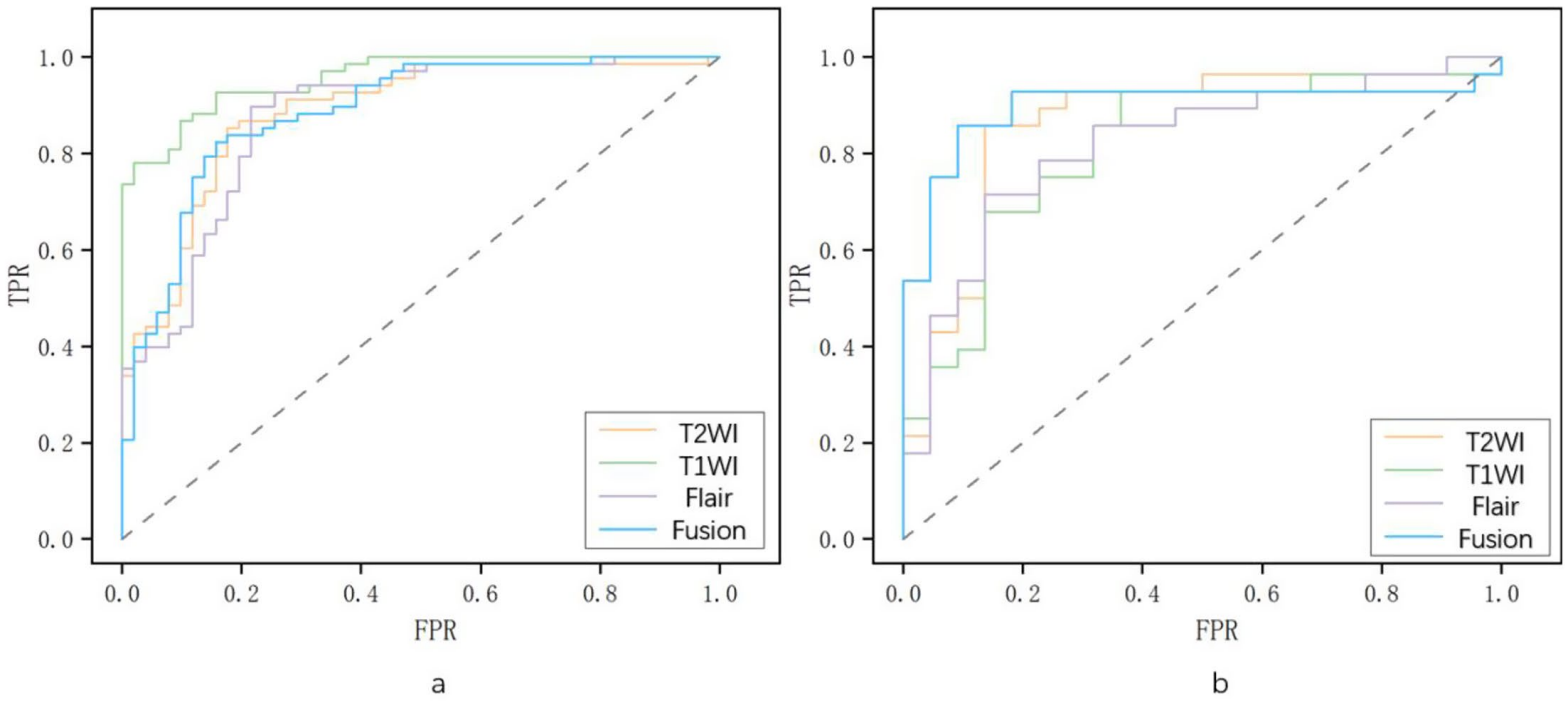
The area under the ROC curve of for different models. **(a)** Training set and **(b)** test set. The gray diagonal lines indicate an AUC value of 0.5, which means the prediction result of completely random.

Three T1WI-, T2WI-, and T2-FLAIR-based models and a fusion radiomics model based on the combination of the three sequences were established. It was found that the fusion radiomics model had the best performance, with an AUC of 0.894 (95% CI, 0.799–0.968), accuracy of 82.0%, sensitivity of 92.9%, and specificity of 68.2%. For the T1WI-, T2WI- and T2-FLAIR-based models, the accuracy was 76.0, 84.0 and 76.0%, the sensitivity was 75.0, 92.9 and 75.0%, the specificity was 77.3, 72.7 and 77.2%, and the AUC was 0.816 (95% CI, 0.710–0.912), 0.864 (95% CI, 0.755–0.951) and 0.820 (95% CI, 0.705–0.91), respectively. The statistical selection process of features with the LASSO algorithm is shown in Figure 3. The AUCs of different models are shown in Figure 4.

The image features of the fusion model are summarized as follows: T2_log-sigma-2-mm-3D_grIm_GrayLevelNonUniformityNormalized, FLAIR_wavelet-HLL_gIrlm_ShortRunLowGrayLeveEmphasis, FLAIR_wavelet-LHL_ngtdm_Contrast, T1_original_firstorder_10Percentile, T2_wavelet-LLL_glcm_SumAverage, T2_original_glcm_JointEntropy, T2_log-sigma-3-mm-3D_glcm_SumEntropy, T2_original_glcm_SumAverage, T1 log-sigma-2-mm-3D_firstorder_10Percentile, T2_original_shape_Elongation, T2_original_glcm_JointAverage, T2_wavelet-LLL_glcm_JointAverage.

### 3.3 Radiologists reading

**Figure 5:**
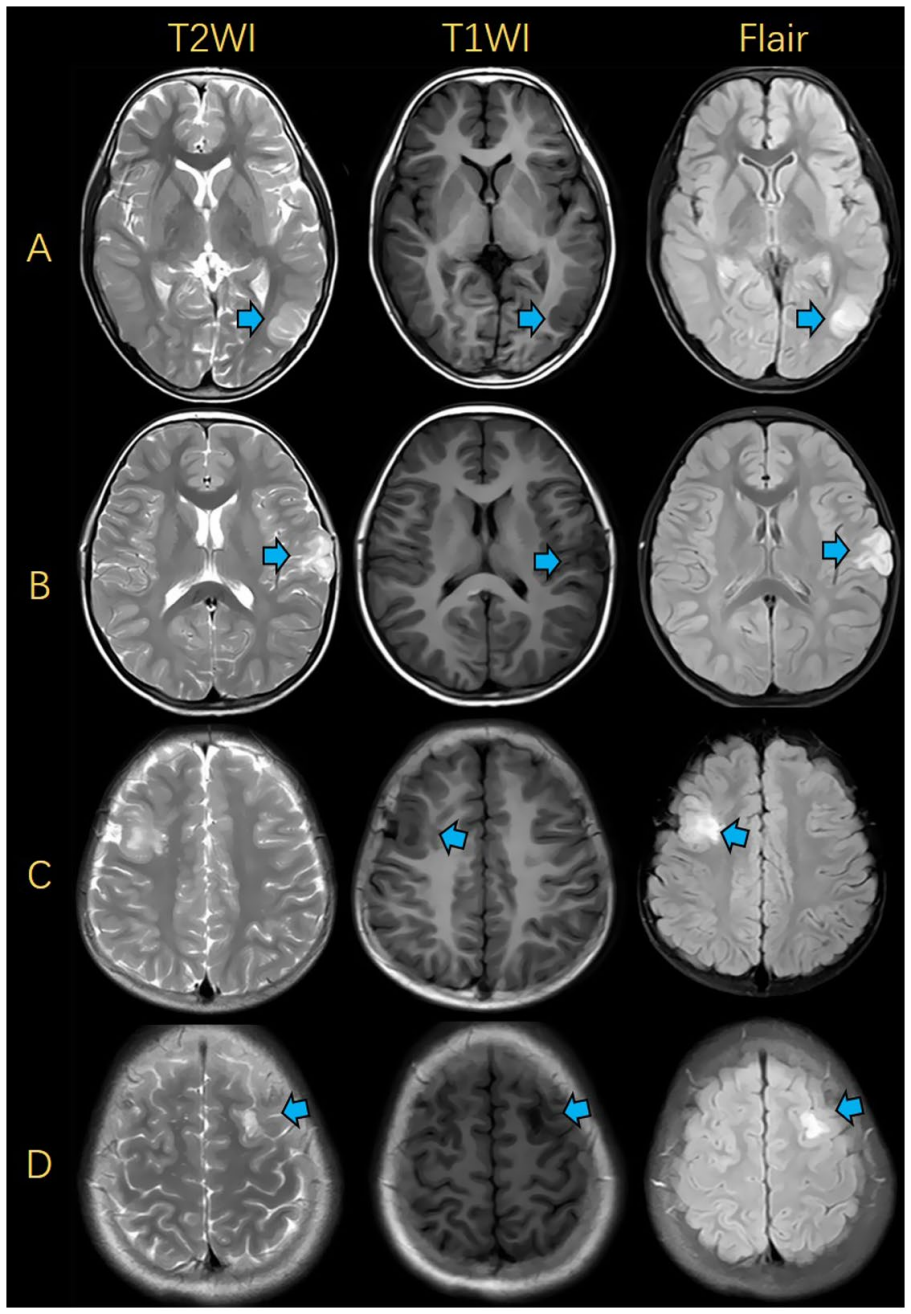
Illustrations of MRIs for FCD and DNET. **(A)** FCD was presented in the left occipital lobe. **(B)** DNET was presented in the left frontal lobe. **(C)** FCD was presented in the right frontal lobe. **(D)** DNET was presented in the left frontal lobe. FCD, focal cortical dysplasia; DNET, dysembryoplastic neuroepithelial tumors.

The accuracy, sensitivity and specificity of the junior radiologist’s assessment were50.0, 53.6, and 45.5%, respectively, while those of the senior radiologist’s assessment were 76.0, 78.6, and 72.7%, respectively. The diagnostic performance of the fusion radiomics model was superior to that of the junior radiologist and comparable to that of the senior radiologist (Table 2). During radiologists reading, there were some easily-confused cases, which led to misdiagnosis. The representative FCD and DNET cases with similar imaging findings are shown in Figure 5.

**Table 2:** Diagnostic performance of comparison of radiomics and human assessment.

Model	Train model				Test model				Radiologist	
	T1WI	T2WI	Flair	Fusion	T1WI	T2WI	Flair	Fusion	Junior	Senior
AUC (95%CI)	0.956 [0.926–0.981]	0.885 [0.832–0.938]	0.875 [0.811–0.926]	0.884 [0.826–0.938]	0.816 [0.710–0.912]	0.864 [0.755–0.951]	0.820 [0.705–0.910]	0.894 [0.799–0.968]		
Accuracy	86.6%	83.2%	84.0%	79.0%	76.0%	84.0%	76.0%	82.0%	50.0%	76.0%
Sensitivity	92.6%	91.2%	92.6%	88.2%	75.0%	92.9%	75.0%	92.9%	53.6%	78.6%
Specificity	78.4%	72.5%	72.5%	66.7%	77.3%	72.7%	77.2%	68.2%	45.5%	72.7%
Youden index	71.1%	63.7%	65.2%	55.0%	52.3%	65.6%	52.3%	61.0%		

AUC, area under the curve; CI, confidence interval.

## 4 Discussion

In this study, several MRI-based radiomics models were established to differentiate FCD from DNET. Among them, the fusion radiomics model exhibited the highest prediction accuracy, with an AUC of 0.894. Importantly, the diagnostic performance of the fusion radiomics model was superior to that of the junior radiologist and comparable to that of the senior radiologist, suggesting its clinical application value. To the best of our knowledge, this is the first time that an MRI-based radiomics model is used for the differential diagnosis of FCD and DNET, the two most common epileptogenic lesions in children. Given the different surgical strategies and prognosis between FCD and DNET, this model is expected to provide a new method for preoperative differential diagnosis.

A total of 12 features were extracted in this study, mainly including 2 first-order features, 11 shape features, 6 gray-level co-occurrence matrix (GLCM) features, 2 gray-level run-length matrix (GLRLM) features, 4 wavelet features, and 3 log-sigma features. The shape features are used to describe the geometric features of the image, providing quantifiable indicators for morphological analysis of tumors. GLCM features, GLRLM features, and log-sigma features reflect the image texture, describing the pixel spatial distribution. Since texture can make full use of image information, it serves as an important basis for image description and recognition. It is challenging to recognize these features by visual perception, but they offer valuable insights into the tumor cell structure and microenvironment, providing powerful evidence for differentiation of DNET and FCD (21). Texture outperforms other image features by effectively capturing both the global properties and intricate details of the image. The wavelet features can effectively capture the image texture information of various scales, thereby providing valuable information for the differentiation and classification of lesions. They exhibit a strong ability of prognostic evaluation and serve as crucial components in radiomics modeling (22). In addition, first-order features are used to describe the distribution of grayscale value within an image for the differentiation and classification of lesions.

Radiomics features have been widely used in radiomics research, which display good performance in the differentiation of lesions. They combine many features of the image and allow a more comprehensive analysis, resulting in higher sensitivity and accuracy in lesion diagnosis than a few or a single image indicator (18). The concept of radiomics originates from medical imaging but surpasses it in terms of complexity and scope. It has drawn great attention in various domains such as image recognition and data analysis. Transforming the image into multidimensional quantitative data has emerged as a valuable tool to contribute to clinical decision-making (23), which can differentiate two similar diseases by extracting their features, greatly improving preoperative diagnostic accuracy and providing a useful reference for surgical planning. In this study, the radiomics model exhibited superior performance in differentiating between FCD and DNET. This model makes accurate preoperative differentiation of FCD and DNET among epilepsy patients feasible, allowing clinicians to effectively tailor surgical plans for better outcomes and quality of life.

Our research ultimately identified these 12 features as describing a highly heterogeneous tumor image: Firstly, multiple texture features such as GrayLevelNonUniformity, Contrast, JointEntropy, and SumEntropy all point to a chaotic internal structure of the tumor, possibly containing various components (such as active tumor, necrosis, and edema); Secondly, multiple intensity-related features on T2 and FLAIR, such as SumAverage, JointAverage, and ShortRunLowGrayLevelEmphasis, suggest that there may be extensive edema and necrotic areas within the tumor; Thirdly, the `Elongation` feature indicates that the tumor has an irregular shape and grows in an infiltrative manner rather than in a clearly demarcated expansive way. Finally, the features are derived from multiple sequences including T1, T2, and FLAIR, and even different processing methods of the same sequence (original, wavelet, LoG filtering), which allows the model to comprehensively evaluate the two diseases from multiple perspectives (cell density, water content, edges, and different scales), suggesting that FCD and DNET have completely different pathological features internally. This radiomics model can help distinguish between these two diseases, which is crucial for the selection of treatment plans and the assessment of treatment effects.

In addition, radiomics can serve as a supplementary approach to certain challenges encountered by radiologists. Frequent work interruption is associated with increased time consumption and an increased likelihood of errors (24). Moreover, some cognitive biases may adversely affect the diagnostic accuracy (25). Radiomics possesses significant advantages in reducing reporting time and cognitive biases, especially for junior radiologists lacking relevant experience (26). However, current radiomics strategies involve excessive post-processing before establishing appropriate machine learning models, and more studies on the effect-cost balance of such machine learning systems are needed before their clinical application.

In previous studies on childhood diseases, MR sequences with long scanning time were used (27), which was quite time-consuming and harmed the physical and mental health of patients. For younger children who cannot fully cooperate, there are certain difficulties in image acquisition, and the image quality cannot meet the requirements. Considering the high dose of anesthetic drugs, the impact on the brain development of children cannot be estimated. Besides, the longer the scanning time, the worse the compliance of children with claustrophobia, and the image quality also presents difficulties to radiologists and even fails to meet the diagnostic requirements (28).

For the above reasons, conventional MRI images were used in this study, which not only meet ethical requirements but also reduce the physical and mental burden of children.

To the best of our knowledge, few studies are available on the differential diagnosis of FCD and DNET in child patients with epilepsy. Previous studies using DTI parameters to evaluate the changes of white matter surrounding epileptogenic foci suggested that only mean diffusivity may be useful in the differentiation of DNET from FCD, and this conclusion was based on the premise that the lesions showed hyperintensity on T2 and T2-FLAIR (29), which largely limited the inclusion of cases. The findings of this study may provide a reference for future research on this population. In the case of FCD, neurosurgeons can perform wide cortical resection over the MRI-delineated lesion, which can help reduce the incidence of postoperative epilepsy in patients with FCD. Furthermore, having a thorough understanding of the possible postoperative outcomes may increase the doctor-patient trust during epilepsy surgery.

This study still had some limitations. Firstly, the sample size was small, and the dataset was collected from one local tertiary hospital. In the future, a prospective multicenter study with a larger sample size should be conducted. Secondly, only T1WI, T2WI, and T2-FLAIR images were used, so multi-modal imaging data need to be included in the future, such as diffusion-weighted imaging (DWI) and arterial spin labeling. Thirdly, the diagnostic efficacy of radiomics models is related to the fusion features, so more features should be considered to achieve better performance.

In conclusion, a cost-effective, convenient, and non-invasive fusion radiomics model was established based on multiple features from conventional MRI images (T1WI, T2WI, and T2-FLAIR) to effectively differentiate between FCD and DNET in child patients with epilepsy before surgery. This model holds significant potential as a valuable reference for surgical planning and contributes to clinical practice.

## Data Availability

The datasets presented in this article are not readily available because due to the limitations of the ethics committee database. Requests to access the datasets should be directed to Xinyi Yang; xinyimsu@163.com.

## Footnotes

^1^ http://fsl.fmrib.ox.ac.uk/fsl/fslwiki/FSL

## References

[1] Bast T Ramantani G Seitz A Rating D. Focal cortical dysplasia: prevalence, clinical presentation and epilepsy in children and adults. Acta Neurol Scand. (2006) 113:72–81. doi: 10.1111/j.1600-0404.2005.00555.x, PMID: 16411966

[2] Piao YS Lu DH Chen L Piao Y‐S Lu D‐H Liu J . Neuropathological findings in intractable epilepsy: 435 Chinese cases. Brain Pathol. (2010) 20:902–8. doi: 10.1111/j.1750-3639.2010.00386.x, PMID: 20331616 PMC8094631

[3] Luzzi S Elia A Del Maestro M Elbabaa SK Carnevale S Guerrini F . Dysembryoplastic Neuroepithelial tumors: what you need to know. World Neurosurg. (2019) 127:255–65. doi: 10.1016/j.wneu.2019.04.056 30981794

[4] Kim DW Lee SK Chu K Park KI Lee SY Lee CH . Predictors of surgical outcome and pathologic considerations in focal cortical dysplasia. Neurology. (2009) 72:211–6. doi: 10.1212/01.wnl.0000327825.48731.c3, PMID: 19005176

[5] Englot DJ Berger MS Barbaro NM Chang EF. Factors associated with seizure freedom in the surgical resection of glioneuronal tumors. Epilepsia. (2012) 53:51–7. doi: 10.1111/j.1528-1167.2011.03269.x, PMID: 21933181

[6] Krsek P Maton B Jayakar P Dean P Korman B Rey G . Incomplete resection of focal cortical dysplasia is the main predictor of poor postsurgical outcome. Neurology. (2009) 72:217–23. doi: 10.1212/01.wnl.0000334365.22854.d3, PMID: 19005171

[7] O'Brien DF Farrell M Delanty N Traunecker H Perrin R Smyth MD . The children's Cancer and Leukaemia group guidelines for the diagnosis and management of dysembryoplastic neuroepithelial tumours. Br J Neurosurg. (2007) 21:539–49. doi: 10.1080/02688690701594817, PMID: 18071981

[8] Rácz A Müller AM Schwerdt J Becker A Vatter H Elger CE. Age at epilepsy onset in patients with focal cortical dysplasias, gangliogliomas and dysembryoplastic neuroepithelial tumours. Seizure. (2018) 58:82–9. doi: 10.1016/j.seizure.2018.04.002, PMID: 29677585

[9] Rheims S Rubi S Bouvard S Bernard E Streichenberger N Guenot M . Accuracy of distinguishing between dysembryoplastic neuroepithelial tumors and other epileptogenic brain neoplasms with [^11^C]methionine PET. Neuro-Oncology. (2014) 16:1417–26. doi: 10.1093/neuonc/nou022, PMID: 24598358 PMC4165411

[10] Gillies RJ Kinahan PE Hricak H. Radiomics: images are more than pictures, they are data. Radiology. (2016) 278:563–77. doi: 10.1148/radiol.2015151169, PMID: 26579733 PMC4734157

[11] Yip SS Aerts HJ. Applications and limitations of radiomics. Phys Med Biol. (2016) 61:R150–66. doi: 10.1088/0031-9155/61/13/R150, PMID: 27269645 PMC4927328

[12] Gombolay GY Gopalan N Bernasconi A Nabbout R Megerian JT Siegel B . Review of machine learning and artificial intelligence (ML/AI) for the pediatric neurologist. Pediatr Neurol. (2023) 141:42–51. doi: 10.1016/j.pediatrneurol.2023.01.004, PMID: 36773406 PMC10040433

[13] Bi WL Hosny A Schabath MB Giger ML Birkbak NJ Mehrtash A . Artificial intelligence in cancer imaging: clinical challenges and applications. CA Cancer J Clin. (2019) 69:127–57. doi: 10.3322/caac.21552, PMID: 30720861 PMC6403009

[14] Huang TM Kecman V. Gene extraction for cancer diagnosis by support vector machines--an improvement. Artif Intell Med. (2005) 35:185–94. doi: 10.1016/j.artmed.2005.01.006, PMID: 16026974

[15] Qian Z Li Y Wang Y Li L Li R Wang K . Differentiation of glioblastoma from solitary brain metastases using radiomic machine-learning classifiers. Cancer Lett. (2019) 451:128–35. doi: 10.1016/j.canlet.2019.02.054, PMID: 30878526

[16] Han H Jiang X. Overcome support vector machine diagnosis overfitting. Cancer Inform. (2014) 13:145–58. Published 2014 Dec 9. doi: 10.4137/CIN.S13875, PMID: 25574125 PMC4264614

[17] Tan YL Kim H Lee S Tihan T ver Hoef L Mueller SG . Quantitative surface analysis of combined MRI and PET enhances detection of focal cortical dysplasias. NeuroImage. (2018) 166:10–8. doi: 10.1016/j.neuroimage.2017.10.065, PMID: 29097316 PMC5748006

[18] Moons KG Altman DG Reitsma JB Ioannidis JP Macaskill P Steyerberg EW . Transparent reporting of a multivariable prediction model for individual prognosis or diagnosis (TRIPOD): explanation and elaboration. Ann Intern Med. (2015) 162:W1–W73. doi: 10.7326/M14-0698, PMID: 25560730

[19] Qian Z Zhang L Hu J Chen S Chen H Shen H . Machine learning-based analysis of magnetic resonance Radiomics for the classification of Gliosarcoma and glioblastoma. Front Oncol. (2021) 11:699789. doi: 10.3389/fonc.2021.699789, PMID: 34490097 PMC8417735

[20] Blümcke I Thom M Aronica E Armstrong DD Vinters HV Palmini A . The clinicopathologic spectrum of focal cortical dysplasias: a consensus classification proposed by an ad hoc task force of the ILAE diagnostic methods commission. Epilepsia. (2011) 52:158–74. doi: 10.1111/j.1528-1167.2010.02777.x, PMID: 21219302 PMC3058866

[21] Gore S Chougule T Jagtap J Saini J Ingalhalikar M. A review of Radiomics and deep predictive modeling in glioma characterization. Acad Radiol. (2021) 28:1599–621. doi: 10.1016/j.acra.2020.06.016, PMID: 32660755

[22] Park CJ Choi YS Park YW Ahn SS Kang SG Chang JH . Diffusion tensor imaging radiomics in lower-grade glioma: improving subtyping of isocitrate dehydrogenase mutation status. Neuroradiology. (2020) 62:319–26. doi: 10.1007/s00234-019-02312-y, PMID: 31820065

[23] Lambin P Rios-Velazquez E Leijenaar R Carvalho S van Stiphout RGPM Granton P . Radiomics: extracting more information from medical images using advanced feature analysis. Eur J Cancer. (2012) 48:441–6. doi: 10.1016/j.ejca.2011.11.036, PMID: 22257792 PMC4533986

[24] Williams LH Drew T. Distraction in diagnostic radiology: how is search through volumetric medical images affected by interruptions? Cogn Res Princ Implic. (2017) 2:12. doi: 10.1186/s41235-017-0050-y, PMID: 28275705 PMC5318487

[25] Lee CS Nagy PG Weaver SJ Newman-Toker DE. Cognitive and system factors contributing to diagnostic errors in radiology. AJR Am J Roentgenol. (2013) 201:611–7. doi: 10.2214/AJR.12.10375, PMID: 23971454

[26] Thrall JH Li X Li Q Cruz C do S Dreyer K . Artificial intelligence and machine learning in radiology: opportunities, challenges, pitfalls, and criteria for success. J Am Coll Radiol. (2018) 15:504–8. doi: 10.1016/j.jacr.2017.12.026, PMID: 29402533

[27] Colon AJ van Osch MJ Buijs M Grond JV Boon P van Buchem MA . Detection superiority of 7 T MRI protocol in patients with epilepsy and suspected focal cortical dysplasia. Acta Neurol Belg. (2016) 116:259–69. doi: 10.1007/s13760-016-0662-x, PMID: 27389578 PMC4989014

[28] Goo HW. Whole-body MRI in children: current imaging techniques and clinical applications. Korean J Radiol. (2015) 16:973–85. doi: 10.3348/kjr.2015.16.5.973, PMID: 26355493 PMC4559794

[29] Rau A Kellner E Foit NA Lützen N Heiland DH Schulze-Bonhage A . Discrimination of epileptogenic lesions and perilesional white matter using diffusion tensor magnetic resonance imaging. Neuroradiol J. (2019) 32:10–6. doi: 10.1177/1971400918813991, PMID: 30461353 PMC6327366

